# Epidemiological Analysis of the COVID-19 Clusters in the Early Stages of the Epidemic in Shanghai, China: Pandemic-to-Epidemic Response Shift

**DOI:** 10.3390/tropicalmed10060170

**Published:** 2025-06-17

**Authors:** Dechuan Kong, Qiwen Fang, Jian Chen, Linjie Hu, Yihan Lu, Yaxu Zheng, Yiyi Zhu, Bihong Jin, Wenjia Xiao, Shenghua Mao, Chenyan Jiang, Xiaohuan Gong, Sheng Lin, Ruobing Han, Xiao Yu, Qi Qiu, Xiaodong Sun, Hao Pan, Huanyu Wu

**Affiliations:** 1 Department of Infectious Diseases Control and Prevention, Shanghai Municipal Center for Disease Control and Prevention, Shanghai 200336, China; 2 Department of Epidemiology, School of Public Health, Fudan University, Shanghai 200032, China; 3 Key Laboratory of Public Health Safety, School of Public Health, Fudan University, Shanghai 200032, China; 4 Institute of Medical Research, Huashan Hospital, Fudan University, Shanghai 200032, China; 5 Office of the Director, Shanghai Municipal Center for Disease Control and Prevention, Shanghai 200336, China

**Keywords:** COVID-19, clusters, epidemiology, transmission, outbreak

## Abstract

As COVID-19 transitions from pandemic to endemic, our prevention and control policies have shifted from broad, strict community interventions to focusing on the prevention of cluster outbreaks. Currently, information on the characteristics of cluster outbreaks remains limited. This study describes the features of COVID-19 clusters in Shanghai. It aims to provide valuable insights for managing localized outbreaks. We conducted a retrospective analysis of clusters of confirmed COVID-19 cases. Epidemiological descriptions, the transmission characteristics of clusters, and individual risk factors for contagiousness were analyzed. A total of 381 cases of COVID-19 were confirmed and 67 clusters were identified. Most clusters (58.21%, 39/67) only had two cases, with a declining proportion held by clusters of more cases. Familial transmission was predominant, accounting for 79.10% (53/67) of clusters. Although other types of cluster outbreaks, such as those in workplaces (1.49%, 1/67), occur less frequently compared to household clusters, they tend to involve larger scales and more cases. Workplaces and similar venues are more likely to experience large-scale cluster outbreaks. Contagiousness was higher among cases with runny nose (risk ratio [RR]: 4.8, 95% CI: 1.40–16.44, *p*-value = 0.01) and those with diabetes (RR: 3.8, 95% CI: 1.01–14.60, *p*-value = 0.05). In conclusion, household cluster outbreaks, in particular, are both a key priority and a foundational issue. Establishing an indicator system based on the transmissibility of cases holds significant practical value for infectious disease prevention and control. By enhancing household hygiene and developing a case classification and management system based on transmissibility, it is possible to better prevent and control regional COVID-19 outbreaks.

## 1. Introduction

Achieving the standards to be considered the first Disease X [1,2], the coronavirus disease 2019 (COVID-19) caught humanity off guard in 2019. To provide evidence for improving the global pandemic preparedness system, it is still necessary to map out certain vital lessons we can learn from COVID-19 at the current time [3,4], such as the response to the manifestations of the clusters in the early stages. On the other hand, although the virus is in the post-pandemic period currently, the reality is that it is still circulating globally [5,6,7,8]. Particularly as it shifts from a pandemic to an endemic phase [9,10,11,12,13,14], localized outbreaks, especially in households and densely populated areas, have become the focus of current prevention and control efforts.

Clusters of COVID-19 cases have been reported worldwide [15,16,17,18,19]. Xinfadi Market outbreak in Beijing, China, the Daegu gathering of Shincheonji Church in South Korea, Japan’s Diamond Princess Cruise Ship Cluster Outbreak and the Smithfield pork processing plant outbreak in the United States have drawn significant global attention [20,21,22,23]. Due to factors such as super-spreader events and pathogen mutations, cluster outbreaks are highly prone to occur in crowded collective settings [24,25,26,27,28]. These outbreaks are characterized by high containment difficulty, prolonged duration, and substantial resource demands, making them a key driver of epidemic escalation.

In the post-pandemic era of COVID-19, epidemic prevention and control strategies require urgent transformation and upgrading [7,29,30]. Preventing cluster outbreaks in key locations has become the focus of COVID-19 prevention and control efforts [31]. The timely and effective containment of cluster outbreaks have become particularly crucial. Failure to promptly control small-scale cluster outbreaks may potentially escalate into cross-border, large-scale transmission risks [32]. Shanghai’s implementation of the “Four Early” strategy (early detection, reporting, isolation, and treatment) for COVID-19 containment proved highly effective, with the incidence rates among its registered population reaching remarkably low levels of 1.64 per 100,000 in 2020 and further declining to 0.65 per 100,000 in 2021 [33,34]. These figures represent significantly lower transmission rates compared to other global metropolises such as New York and Singapore during the same period [35].

Information regarding the epidemiological and clinical characteristics of COVID-19 clusters is limited. This study analyzes the characteristics of cluster outbreaks during the early stages of COVID-19 in Shanghai and compares the transmissibility based on clinical and health care-related risk factors. It aims to provide valuable insights for improving preparedness of emerging respiratory infectious diseases and managing current localized outbreaks.

## 2. Materials and Methods

### 2.1. Study Design and Data Source

At the beginning of the pandemic, most cases had a traceable source of infection. In the later stages of the pandemic, as the virus spread widely, tracking cluster outbreaks became extremely challenging. In this observational study, we conducted a retrospective analysis of clusters of confirmed COVID-19 cases that were diagnosed and reported through the China Information System for Disease Control and Prevention before October 2021 in the early stages of the epidemic in Shanghai.

According to the cluster investigation technical guidelines in China, a cluster is defined as ≥2 confirmed cases or asymptomatic infected persons found in a small area (such as households, workplaces, vehicles, etc.) within 14 days through epidemiological investigation. This definition considers the possibility of human-to-human transmission caused by close contact or exposed to a common infection source.

Individual data from investigation reports through interviews by CDC staff and medical records were systematically entered and managed in EpiData version 3.1 (EpiData Association, Odense, Denmark). Cases were coded by unique identification numbers and were de-duplicated prior to analysis. Variables of interest included demographic information (age, gender, occupation), initial symptoms at onset (self-reported by the cases during epidemiological investigation), the comorbid condition (including body mass index (BMI)), severity of illness, and indicators at first clinical visit. Data processing was conducted in accordance with observational study principles.

### 2.2. Statistical Analysis and Study Definitions

#### 2.2.1. Epidemiology of Clusters

We graphed epidemic curves of clusters by both date of illness onset and date of report. Time of cluster formation was defined as the time when the second case involved fell ill or was identified. Key dates relating to important events were also labelled to explore their influence on the spread of the outbreak.

#### 2.2.2. Analysis on the Transmission Features of Clusters

Using information gleaned about the relationship between clustered cases, we categorized the clusters into 4 types: familial, occupational, social and mixed.

Analysis of the serial interval (i.e., the average days between onset of cases in a chain of transmission) was conducted based on explicit chains we selected to make sure the transmissions were undisputed. All secondary cases should meet the following criteria for exposures occurring within 1–14 days before the onset of illness: (a) contact with exactly one primary case; (b) no history of trips or residency related to Wuhan and surrounding areas or other communities where confirmed cases existed; (c) no other potential exposures, such as hospital visits, etc. R_e_ was calculated using Rpackage R_e_.

Cases were classified as either (1) contagious with confirmed secondary transmission, or (2) non-contagious. Based on the principles of statistical model selection, we employed log-binomial regression models to directly estimate risk ratios (RRs), which is statistically preferable for binary outcomes with common event frequencies (>10%) compared to logistic regression [36]. Each risk factor was evaluated in a separate model adjusted for age and sex as core confounders, following the parsimony principle to avoid multicollinearity and overfitting given the exploratory nature of this analysis. Variables with fewer than 5 contagious cases were excluded to ensure model stability. The Holm–Bonferroni method was applied to correct for multiple testing. Significance was assessed at an α = 0.05 level. Data were analyzed in SAS version 9.4 (SAS Institute, Cary, NC, USA). Figures in the study were processed by R (v4.0.4, R Foundation for Statistical Computing, Vienna, Austria. URL https://www.R-project.org/, accessed on 5 June 2025) and Excel 2016 (Microsoft Office, Redmond, WA, USA).

### 2.3. Ethics Approval

This study was reviewed and approved by the Shanghai Municipal CDC Ethical Review Committee. Patients were not given an informed consent form as the data were collected as part of ongoing public health surveillance.

## 3. Results

A total of 381 cases of COVID-19 were reported in Shanghai between January 2020 and October 2021. Most (61.42%, 234/381) of the cases occurred in clusters, with 67 clusters being reported in this time period. Most clusters (58.21%, 39/67) only had 2 cases, with a declining proportion held by clusters of more cases: 14.92% (10/67), 11.94% (8/67) and 22.39% (15/67) for clusters of 3, 4, and ≥5 cases, respectively.

### 3.1. Epidemiological Description of Clusters in Shanghai

**Figure 1:**
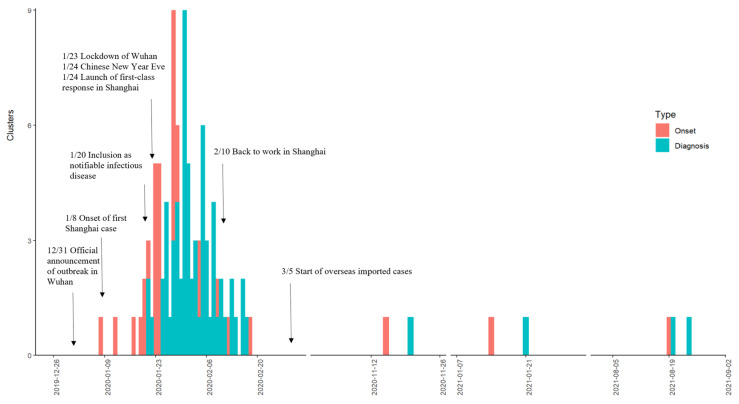
Timelines and characteristics of 67 COVID-19 clusters in the early stages of the epidemic in Shanghai.

By the date of onset, clusters began to occur sporadically from January 3, reaching a peak on January 29 and then gradually decreasing. By the date of report, clusters began to be identified on January 21 and then declined after reaching the peak on January 31. We found the report peak occurred about 2 days after the onset peak. After January 24 (Chinese New Year Eve), the Shanghai government launched a first-class response, and a decline in the clusters was observed about 7 days later by the onset date. After considering the increased risk of COVID-19 transmission during the massive human migration across the provinces that occurs during the holiday (January 24–February 2), the Chinese national government delayed the start of work to February 10 (Figure 1).

We have identified a total of 67 clusters, involving 234 cases. A total of 130 cases (55.56%, 130/234) were locally infected in Shanghai, and 89 cases (38.03%, 89/234) were infected in five other provinces in China; 8 cases (3.42%, 8/234) were infected in foreign countries (5 in Europe and 3 in Asia); the locations of infection for 7 cases (2.99%, 7/234) were not able to be determined. Of the 104 cases who were infected outside Shanghai, 33.65% (35/104) arrived in Shanghai by aircraft, 28.85% (30/104) by high-speed railway/bullet train, and 25.00% (26/104) by driving private cars.

### 3.2. Transmission Features of Clusters

**Figure 2:**
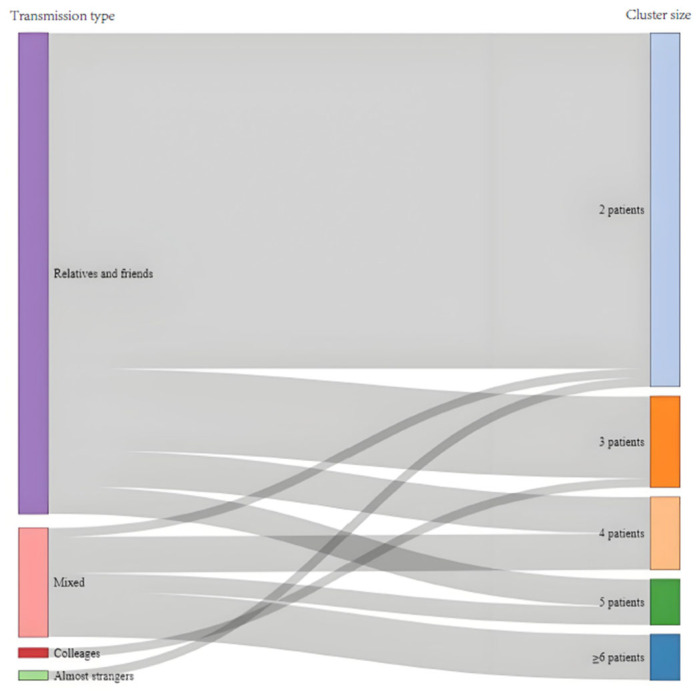
Transmission features of clusters in Shanghai. This Sankey diagram visualizes the relationship between transmission type and cluster size observed in the early stage of COVID-19 pandemic in Shanghai. Left side refers to the contact pattern and social relations of the cases in the cluster, including relatives and friends, colleagues, almost strangers, and combination of these patterns (mixed). Right side refers to the number of patients involved in the cluster. The gray bands show each connection indicating the cluster size of the certain transmission type.

An analysis of the relationship among cases involved in the 67 clusters revealed that familial transmission (“Relatives and friends” in Figure 2) was predominant, accounting for 79.10% (53/67) of clusters. Exposure in workplaces (“Colleagues” in Figure 2) was responsible for one cluster of three cases. One cluster (1.49%, 1/67) occurred on the same tour group (“Almost strangers” in Figure 2), involving two cases. Additionally, 12 clusters (17.91%, 12/67) (“Mix” in Figure 2) were not able to be categorized into one type of cluster; all these mixed clusters involved familial transmission.

The serial interval was calculated for 33 clear transmission chains from 67 clusters, excluding 2 chains with equivocal onset time. We found that the serial interval in Shanghai ranged from −1.83 to 14.63, and the median and IQR were 5.50 (2.47, 11.47) during the early stages (Figure 1). R_e_ was calculated as 2.9 (95% CI: 2.50–3.29) using the exponential growth method and 2.6 (95% CI: 2.07–3.14) using the maximum likelihood method. Two peaks emerged at day 1–3 and day 11–13. Two chains with onset of subsequent generations earlier than the last generation (1.83, 0.50 days) were observed, indicating transmission during the incubation period. We observed a large variability in serial interval when two generations met 1–2 times instead of having continuous exposure (≥3 or more days, such as when living together), i.e., 14.63 days (contacted twice), 12.63 days (contacted once), 12.46 days (contacted once), 2.08 days (contacted once) and 1.63 days (contacted once).

### 3.3. Individual Risk Factors of Contagiousness

We separated individuals into those who were non-contagious cases and those contagious, i.e., having successive generations (Table 1). Contagiousness was higher among cases with a sore throat (risk ratio [RR]: 2.9) and runny nose (RR: 4.8), and those with diabetes (RR: 3.8). Delays in diagnosis were also associated with higher risk of contagiousness. Having ≥2 medical visits before diagnosis was associated with a 1.1-time higher risk of contagiousness.

## 4. Discussion

Our study characterizes COVID-19 clustered outbreaks, revealing that small-scale, family-based transmission predominates. Larger-scale clustered outbreaks were more likely to occur in workplace and public settings. Epidemiological analysis identified key secondary transmission risk factors, including index cases presenting with a sore throat or rhinitis, pre-existing diabetes, and delays in diagnosis/isolation. Using the early stages surveillance data, we provide empirical evidence that advances our understanding of transmission heterogeneity. Significantly, we propose a novel transmissibility-based classification system with direct applications for optimizing outbreak response strategies.

From the perspective of the outbreak cluster scale, cluster outbreaks exhibit substantial variation in scale, ranging from just a few cases to thousands of infections; super-spreading events, in particular, tend to trigger outbreaks of a significantly larger scale [16,37,38,39,40]. In our study, the overall scale of outbreaks was relatively small. This pattern resembles the scale characteristics of cluster outbreaks observed in Sichuan and Jilin during China’s early epidemic phase [17,41], which may be attributed to their occurrence during the early stages of the epidemic and the timely detection and containment measures implemented. Regardless of the outbreak scale, inadequate containment measures may escalate localized clusters into large-scale epidemics. Although vaccines demonstrate significant efficacy, their infection-blocking capacity remains limited [42,43,44,45,46,47]. Multiple factors influence the scale of outbreaks, including environmental conditions at the exposure site, population immunity levels, and pathogen variants. Additionally, in each outbreak event, symptomatic cases are more likely to be detected and reported than asymptomatic infections [48,49,50,51,52]. During the pandemic phase, cluster outbreaks consistently produced high numbers of symptomatic cases, particularly in the pre-Omicron period and in regions with low vaccination coverage [48,53,54,55,56,57,58]. In contrast, the post-pandemic (May 2023 onward) phase is characterized by outbreaks that primarily result in asymptomatic or mild infections [8,59,60,61]. This epidemiological shift necessitates complementary strategies beyond vaccination, including active surveillance systems to detect asymptomatic infections—especially in high-risk congregate settings such as nursing homes and schools where vulnerable populations are concentrated.

**Figure 3:**
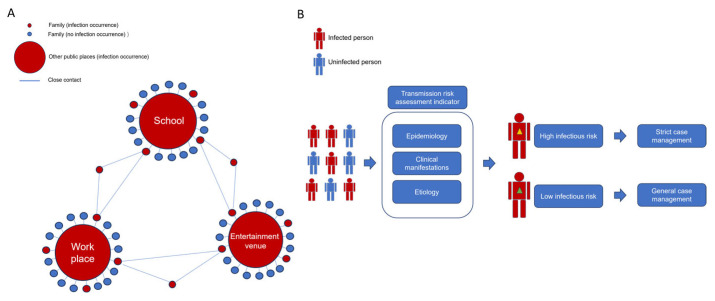
Conceptual diagram (**A**): The key bridging role of families in the transmission of COVID-19. The schematic employs a standardized visual encoding system where (1) chromatic coding distinguishes infection status (red = infected, blue = uninfected), (2) topological scaling represents outbreak magnitude (circle diameter = case count), and (3) geometric relationships depict transmission pathways. Specifically: small red circles denote infected family, large red circles indicate infected public venues, blue circles represent unaffected locations, and blue lines trace close-contact transmission chain. (**B**): Classification and management of COVID-19-infected individuals based on the transmission risk assessment indicator system. The schematic employs a dual-coding system to visualize transmission dynamics: (1) chromatic differentiation (red human figures = infected, blue human figures = uninfected), and (2) transmission risk stratification (yellow triangle = high-risk cases requiring strict containment measures; green triangle = low-risk cases managed measures).

In terms of transmission cluster categories, our findings demonstrate that household transmission constitutes a major proportion of COVID-19 clusters and serves as a critical driver of sustained community spread, underscoring the imperative for targeted household-level containment measures. These outbreaks demonstrate remarkable diversity in their nature. Our research findings indicate that in cluster outbreaks of COVID-19, family-based clusters occur frequently (79.10% of clusters) but typically involve a small number of individuals, primarily two. In contrast, clusters associated with social activities or collective settings tend to involve a larger number of cases. This observation aligns with reported studies [15,16,17,19,41,62]. Another study in Paris in 2020 identified educational settings as the primary venue for cluster transmission, representing 54.7% of outbreaks [63]. However, a concurrent UK study showed that educational institutions were not the primary sites of clustered outbreaks, suggesting that controlling community transmission is crucial [64]. The key to controlling community transmission lies in managing both intra-household and inter-household spread [65]. Many studies have identified the critical role of family clusters in the spread of epidemics [66,67,68], with in-depth research revealing key influencing factors of such transmission [69,70,71,72,73,74]. Family plays a critical bridging role in the transmission of epidemics, which could be the common scenario of emerging respiratory infectious diseases. Although family cluster outbreaks are generally small in scale, the occurrence of numerous family-based clusters contributes significantly to the rapid spread of the epidemic. Each family represents the smallest societal unit, serving as a critical bridge and catalyst for initiating larger-scale outbreaks (Figure 3). At the endemic stage, implementing adequate household hygiene measures serves as an effective strategy for both COVID-19 containment and the mitigation of other respiratory communicable diseases.

From an epidemiological and transmissibility perspective of the cases, a transmission-potential-based case classification system holds significant value as a supplementary public health tool. Based on the limited sample size from our single-center study, we explored and identified certain characteristics in some cases that indicate a higher risk of transmission to others. This exploratory analysis holds significance especially when we need to take actions before laboratory results are retrieved. Importantly, our findings can be integrated with laboratory pathogen data to provide a more comprehensive evaluation of case transmission potential. Patients with runny noses and comorbid conditions (diabetes) were found to be more contagious and more easily infect others. Similar investigations have been conducted in other studies [41,75]. Currently, cases are primarily categorized based on clinical severity. However, during the endemic phase, we believe it is necessary to classify cases based on their level of infectiousness and particular significance for implementing early interventions in high-risk settings such as nursing homes and educational institutions. By integrating epidemiological characteristics, clinical manifestations, and laboratory evidence, a classification system for case infectiousness should be established. This system would enable enhanced management of such cases during treatment or recovery, with a focus on preventing further transmission (Figure 3).

Compared to the aforementioned studies, the distinguishing features of our research lie in its temporal proximity to Shanghai’s early-stage containment measures and its investigation of transmission risk at the individual level. However, several limitations should be acknowledged. Primarily, our analysis was limited to early-pandemic data rather than complete pandemic-period data, but it is consistent with the methodological patterns observed in existing cluster outbreak literature [16,66,76,77,78,79,80]. Operationally, early-phase investigations allow more robust cluster identification and granular contact tracing—an approach that becomes logistically prohibitive during peak transmission due to case volume [81]. Notably, our findings align substantively with the studies utilizing mid-pandemic (June 2020–January 2022) or later data (August–September 2022) [82,83], suggesting temporal generalizability of these early-phase observations. Second, the information extracted from field investigation was inevitably influenced by recall bias and reporting bias of the respondents. The methodological constraints of observational studies, particularly selection and recall biases, have been widely recognized across numerous published studies [84,85,86]. We implemented mitigation strategies including (1) having trained public health professionals conduct standardized epidemiological investigations focusing on a fixed time window to trace exposures and contacts; (2) enrolling all identified cluster cases from the early epidemic phase without selective inclusion criteria. Additionally, while the log-binomial model appropriately estimates RRs, its main assumptions including linearity in the log scale, common binary outcomes and absence of perfect separation require careful consideration. Although our data met these assumptions and only age and sex were adjusted in the model to avoid multicollinearity, unmeasured confounders may persist [87,88]. The subgroup analyses with small sample sizes yielded wide confidence intervals and significance indicating reduced precision. Therefore, these results should be interpreted as hypothesis-generating in the scenario of the early stage of the epidemic when information was limited, and validated in larger cohorts. Future studies could integrate pathogen genomic data with larger epidemiological surveillance data to conduct more comprehensive analyses.

## 5. Conclusions

The prevention and control of cluster outbreaks remains a critical focus in the post-pandemic period, requiring long-term attention. Family-based cluster outbreaks, in particular, are both a key priority and a foundational issue. Establishing an indicator system based on the transmissibility of cases holds significant practical value for infectious disease prevention and control. By enhancing household hygiene and developing a case classification and management system based on transmissibility, it is possible to better prevent and control regional COVID-19 outbreaks. Also, it could serve as a good example of identifying and preventing disease transmission of emerging respiratory infectious diseases in the early stages.

## Figures and Tables

**Table 1:** Epidemiological and clinical characteristics of sporadic cases and clustered cases.

	Count (col. %)	Contagious Case ^a^ (row %)	Risk Ratio ^b^ (95% CI)	*p*-Value
Age				
0–19 years	15 (4%)	0 (0%)		
20–39 years	122 (32%)	14 (11%)		
40–59 years	132 (35%)	11 (8%)		
60–88 years	112 (29%)	11 (10%)		
Gender				
Male	203 (53%)	16 (8%)		
Female	178 (47%)	20 (11%)		
Initial symptoms				
Fever				
No	146 (38%)	11 (8%)	1.0 (reference)	
Yes	235 (62%)	25 (11%)	1.0 (0.46–2.30)	0.95
Dry cough				
No	259 (68%)	25 (10%)	1.0 (reference)	
Yes	122 (32%)	11 (9%)	1.3 (0.56–2.85)	0.58
Sore throat				
No	347 (91%)	29 (8%)	1.0 (reference)	
Yes	34 (9%)	7 (21%)	2.9 (1.00–8.48)	0.05
Runny nose				
No	359 (94%)	30 (8%)	1.0 (reference)	
Yes	22 (6%)	6 (27%)	4.8 (1.40–16.44)	0.01
Weakness				
No	338 (89%)	31 (9%)	1.0 (reference)	
Yes	43 (11%)	5 (12%)	0.9 (0.29–2.53)	0.79
BMI ^c^				
Underweight	41 (11%)	2 (5%)	1.23 (0.23, 6.66)	0.81
Normal	170 (45%)	14 (8%)	1.00 (reference)	
Overweight	170 (45%)	20 (12%)	1.94 (0.85,4.42)	0.12
Comorbid condition				
Diabetes				
No	352 (92%)	30 (9%)	1.0 (reference)	
Yes	29 (8%)	6 (21%)	3.8 (1.01–14.60)	0.05
High blood pressure				
No	311 (82%)	27 (9%)	1.0 (reference)	
Yes	70 (19%)	9 (13%)	1.9 (0.64–5.60)	0.25
Heart disease				
No	357 (94%)	32 (9%)	1.0 (reference)	
Yes	24 (7%)	4 (17%)	2.0 (0.46–8.37)	0.36
Clinical manifestation				
Mild (non-pneumonia)	24 (6%)	2 (8%)	1.00 (reference)	
Mild (pneumonia)	332 (87%)	28 (8%)	0.63 (0.12, 3.37)	0.59
Severe	9 (2%)	2 (22%)	1.22 (0.12, 12.45)	0.87
Critically severe	16 (4%)	4 (25%)	12.82 (0.81, 203.79)	0.07
Seeking medical help				
Diagnosed at first medical visit	257 (67%)	16 (6%)	1.0 (reference)	
≥2 medical visits before diagnosis ^d^	124 (33%)	20 (16%)	2.1 (1.00–4.58)	0.05

^a^: Contagious case was defined as an infector having successive generations confirmed by epidemiological investigations. ^b^: Models adjusted for age group and gender, but no other risk factor. Only risk factors with ≥5 contagious cases were considered. ^c^: BMI was divided into three categories according to WHO standards: underweight (BMI < 18.5), normal (18.5–24.9) and overweight (≥25). ^d^: Cases with ≥2 medical visits before diagnosis were not identified in primary care for the first time.

## Data Availability

The datasets generated during the current study are not publicly available but are available from the corresponding author on reasonable request.
